# Aging and decreased glomerular filtration rate: An elderly population-based study

**DOI:** 10.1371/journal.pone.0189935

**Published:** 2017-12-19

**Authors:** Regina C. R. M. Abdulkader, Emmanuel A. Burdmann, Maria Lúcia Lebrão, Yeda A. O. Duarte, Dirce M. T. Zanetta

**Affiliations:** 1 University of São Paulo Medical School, São Paulo, Brazil; 2 School of Public Health of the University of São Paulo, São Paulo, Brazil; The University of Manchester, UNITED KINGDOM

## Abstract

**Background:**

Although a reduced glomerular filtration rate (GFR) in old people has been attributed to physiologic aging, it may be associated with kidney disease or superimposed comorbidities. This study aims to assess the prevalence of decreased GFR in a geriatric population in a developing country and its prevalence in the absence of simultaneous diseases.

**Study design and methods:**

This is a cross-sectional study of data from the Saúde, Bem-Estar e Envelhecimento cohort study (SABE study[Health, Well-Being and Aging]), a multiple cohorts study. A multistage cluster sample composed of 1,253 individuals representative of 1,249,388 inhabitants of São Paulo city aged ≥60 years in 2010 was analyzed. The participants answered a survey on socio-demographic factors and health, had blood pressure measured and urine and blood samples collected. GFR was estimated and defined as decreased when <60 mL/min/1.73m^2^. Kidney damage was defined as dipstick-positive hematuria or urinary protein:creatinine > 0.20 g/g.

**Results:**

The prevalence of GFR <60 mL/min/1.73m^2^ was 19.3%. Individuals with GFR <60 mL/min/1.73m^2^ were older (75±1 versus 69±1 years, p<0.001), had lower schooling (18 versus 30% with complete 8-year basic cycle, p = 0.010), and higher prevalence of hypertension (82 versus 63%, p<0.001), diabetes (34 versus 26%, p = 0.021), cardiovascular disease (43 versus 24%, p<0.001) and kidney damage (35% versus 15%, p<0.001). Only 0.7% of the entire studied population had GFR <60 mL/min/1.73m^2^ without simultaneous diseases or kidney damage. Among the individuals with GFR <60 mL/min/1.73m^2^, 3.5% had neither renal damage nor associated comorbidities, whereas among those with GFR ≥60 mL/min/1.73m^2^, 11.0% had none of these conditions. Logistic regression showed that older age, cardiovascular disease and hypertension were associated with GFR<60 mL/min/1.73m^2^.

**Conclusions:**

Decreased GFR was highly prevalent among the geriatric population in a megalopolis of a developing country. It was rarely present without simultaneous chronic comorbidities or kidney damage.

## Introduction

Chronic kidney disease (CKD) is a public health problem worldwide. In a systematic review of population-based studies, the median prevalence of CKD was 7.2% in people aged ≥ 30 years, but it ranged from 23.4% to 35.8% in people aged ≥ 64 years.[1] Among the 26 studies included in the review, 10 were carried out in North America, 8 in Europe, 6 in Asia and 2 in Australia, but no study was conducted in South America.

Data from the National Health and Nutrition Examination Survey (NHANES 2007–2012) showed a CKD prevalence of 5.7%, 8.9% and 33.2% in age groups of 20–39, 40–59 and ≥ 60 years, respectively. [2]

In Brazil, data are lacking regarding the prevalence of CKD, especially in the elderly. In a survey of Brazilian adults participating in CKD prevention campaigns (mean age 45±22 years), 7.3% of the 37,771 urine samples examined showed proteinuria.[3] The estimated prevalence of patients on chronic dialysis in Brazil in 2012 was 503 per million, with 31.9% aged ≥ 65 years.[4]

Old age is considered to be a risk factor for CKD, but the decrease in the glomerular filtration rate (GFR) with aging may be a consequence of the normal aging process and not a sign of disease. Nevertheless, diseases such as diabetes can superimpose on this process, aggravating GFR decline.[5] When a disease superimposes on aging, GFR decline and other signs, such as proteinuria, may appear. The presence of proteinuria increases the morbidity and mortality induced by a decreased GFR.[6]

Distinguishing between an elderly individual with a “normal for age and gender” low GFR and another with the same GFR resulting from kidney disease is very important because treatment of the first condition has not been proven to reduce the risks of cardiovascular events or the progression to end stage renal disease.[7,8]

There are few studies on CKD in the elderly that take this point into account, especially in developing countries, such as Brazil. In São Paulo, the biggest megalopolis of South America, a GFR < 80 mL/min/1.73m^2^ was found in 68% of 81 selected elderly individuals, and microalbuminuria was found in 31% of these individuals.[9] One of the few elderly population-based studies in Brazil (Bambui study, conducted in a small city in Southeast Brazil) showed that 5.1% of the residents aged > 60 years had an increased serum creatinine (SCr) value.[10] Another Brazilian study reported that, among 822 participants aged ≥ 60 years (a simple random sampling among 9,009 elderly inhabitants of Tubarão city enrolled in the Family Healthcare Strategy Program), 112 (13.6%) had an estimated GFR < 60 mL/min/1.73m^2^.[11] None of these studies investigated the presence of kidney disease in individuals with decreased GFR or abnormal SCr.

The objectives of the present study are to estimate the prevalence of decreased GFR in the elderly population of São Paulo city and the frequency of decreased GFR without simultaneous comorbidities.

## Study design and methods

The study was approved by the Human Research Ethics Committee of the School of Public Health, University of São Paulo (OF.COEP 23/10). Participation was voluntary, and written informed consent was obtained.

This was a cross-sectional study conducted with data obtained in 2010 from the Saúde, Bem-Estar e Envelhecimento cohorts study (SABE [Health, Well-Being and Aging]). SABE is a multiple cohort study that started in 2000 and is composed of a probabilistic sample representative of the population of São Paulo city aged ≥ 60 years. The sample was selected by multistage cluster sampling, as described elsewhere. [12–13] Briefly, in the first phase in 2000, census sectors were selected for sampling with probability proportional to the number of dwellings in each sector. In the second phase, the dwellings were selected by simple random sampling, with a minimum of 90 in each sector. In 2006, a second wave was conducted, and a new sample of individuals aged 60–64 years was enrolled, using the same sample procedure, as at that time this age range was not represented. A third follow-up visit was made in 2010 when a new sample of individuals 60–64 years was included. In 2010, 989 elderly individuals who had been included in the study at baseline and 2006 waves were located and agreed to participate in this third phase of the study, which also included new 355 individuals aged 60–64. Among those lost to follow up, 930 had died, 300 were not found or had moved from the city, 21 were institutionalized and 212 refused to participate in the continuation of the study. Weights were re-calculated considering the census sector of each individual using data from the 2010 Census to maintain a representative sample of the elderly population of São Paulo. The total sample included in 2010 was 1344 individuals aged ≥ 60 years which represented 1,338,138 elderly individuals living in São Paulo city.

Data were obtained by an interviewer-administered questionnaire at the participants’ homes and included information on socio-demographic factors, general health and living conditions. The elderly had their cognitive status evaluated by the Mini Mental State Examination (MMSE, validated for the SABE Study). They were assisted by a proxy respondent when they scored below 12. The MMSE has thirteen items that are less dependent upon schooling because the South American elderly population as a whole has a low level of schooling. [14–15] The collection of blood and urine samples, initiated in the third wave, allowed the evaluation of renal function. The collection of these samples and the anthropometric measurements were performed at the participants’ homes.

The following variables were obtained from the questionnaire: 1. Demographics: age (60-69/70-79/80-89/≥90 years), gender (female/male) and ethnic background (Caucasian/others); 2. Socio-economic characteristics: schooling (0-3/4-7/≥8 years) and monetary income [<2/2-4/>4 minimum wage/month, one Brazilian minimum wage was US$290 in 2010]; 3. Self-perceived health conditions and history of select diseases: self-rated health today (good/poor to fair), smoking status (never smoked/ex-smoker/current smoker), history of hypertension (yes/no), history of diabetes (yes/no) and history (past or current) of cardiovascular disease (yes/no heart problem or stroke). The presence of diseases was evaluated using the question “Has any doctor or nurse ever told you that you have…”.

The following anthropometric variables were considered: systolic and diastolic blood pressure (mean of three measurements in the same visit, mmHg); waist circumference (cm), height (m) and weight (kg) measurements. Body mass index was calculated as the weight divided by the square of the height (BMI, kg/m^2^) and was categorized as low (≤22), normal (22>BMI>27) or overweight or obese (≥27).

Urine samples were collected in the morning and kept cold until the dipstick reading to detect the presence of hemoglobin (one + or more was considered positive). The dipstick was read manually by a trained professional following instructions provided by the manufacturer. (Uri-Color check. WAMA Diagnóstica Ltda, São Carlos, SP, Brazil). After the dipstick reading, the urine samples were kept in -20°C freezer until the creatinine and protein were measured. Protein was measured using 3% sulphosalicylic acid, and creatinine was measured by the Jaffé method. A urinary protein:creatinine ratio > 0.20 g/g was classified as abnormal proteinuria.[16]

The following tests were assessed in fasting blood samples: urea (mg/dL), SCr (mg/dL), calcium (mEq/L), phosphorus (mg/dL), albumin (g/dL), uric acid (mg/dL), hemoglobin (g/dL), iron (μg/dL), ferritin (ng/dL), C-reactive protein (CRP, mg/dL), fasting plasma glucose (FPG, mg/dL), glycohemoglobin (HbA1c, %), cholesterol and its HDL and LDL fractions (mg/dL), and triglycerides (mg/dL). All of these tests were performed at the laboratory of INCOR (Heart Institute, School of Medicine, University of São Paulo), which is certified by ISO 9001.

### Evaluation of renal function

The SCr was analyzed using the Jaffé method (reference values: 0.8–1.3 mg/dL for males and 0.6–1.0 mg/dL for females). GFR was estimated by the simplified Modification of Diet in Renal Disease (MDRD) equation using 4 variables and no standardized SCr: GFR = 186 x (SCr, mg/dL)^-1.154^ x (age, years)^-0.203^ x 1.212 (if African-American) x 0.742 (if female).[17] All participants were classified as non-African-American.

A GFR < 60 mL/min/1.73m^2^ was classified as decreased GFR. A positive dipstick test for urine hemoglobin or an abnormal proteinuria (urinary protein:creatinine ratio > 0.20 g/g) was classified as kidney damage.

### Definitions

An individual was considered to have hypertension if he/she self-reported the disease or if the mean of three measurements of arterial blood pressure was > 140/90 mmHg.[18] An individual was considered to have diabetes mellitus if he/she self-reported the disease or if FPG was ≥ 126 mg/dL or HbA1c was ≥ 6.5%.[19] Metabolic syndrome was defined as having at least three of the following criteria: waist circumference ≥ 90 cm for men or ≥ 80 cm for women, triglycerides ≥ 150 mg/dL, HDL cholesterol ≤ 40 mg/dL for males or ≤ 50 mg/dL for females, systolic blood pressure ≥ 130 mmHg or diastolic blood pressure ≥ 85 mmHg, and FPG ≥ 100 mg/dL. [20]

### Statistical analysis

Descriptive analysis was carried out. Categorical data are presented as weighted percentages, and continuous variables, as weighted means and standard errors. Intergroup comparisons were made using Pearson’s chi-square test with the Rao-Scott correction or t-tests.[21] The analysis incorporated the individuals’ weights to correct for the different selection probabilities of participants and for the data to be representative of the population of São Paulo city aged ≥60 years. The survey package from the R 2.15.3 program was used. [22] The package offers procedures for analysis of complex sample inquiries and allows the incorporation of different weights of observations that influence the parameter estimates of the total population and the effect of sampling on variance estimates. The results are expressed as weighted values. A backward stepwise logistic regression was performed using the survey-weighted generalized linear models (svyglm package), with inverse-probability weighting and design-based standard errors. Dependent variable was decreased GFR (reference absence) and independent variables were age (70-79/80-89/≥90 years, reference 60–69 years) and presence of diabetes, hypertension or cardiovascular disease (reference absence), adjusting for sex, schooling and monetary income. The Wald test was used to evaluate the statistical significance of the association between independent and dependent variables. The Hosmer-Lemeshow test was used to evaluate the goodness of fit of the model.

## Results

Of the 1344 individuals answering the questionnaire, 1253 had their blood collected and entered the study to detect decreased GFR (representative of a population of 1,249,388 individuals), and 998 of them had their urine collected and were evaluated for renal damage (representative of 973,324 individuals). Complete data for the 1253 individuals who entered the study of decreased GFR are shown in S1 File. There were no differences between the individuals with and without blood collection. In the comparison of individuals with and without urine collection they were also similar, except that those without urine samples were slightly older (72.3±0.8 versus 69.8±0.8 years, p<0.001) and had fewer years of schooling (20 versus 30% with complete 8-year basic cycle, p = 0.027).

The individuals were predominantly women (60.1%) and Caucasians (58.6%), and 14.6% aged > 80 years. Most of them went to school (88.5%), but only 28% had completed the 8-year basic cycle, and 87.1% had an income < 4 minimum wage/month. Although public health insurance is universal in Brazil, 44.5% had private health insurance. Forty nine percent perceived their health as good or very good, and 50.8% had never smoked. The prevalence of self-reported hypertension (67%) was strikingly higher than the prevalence of other reported health problems, such as diabetes (24.9%) or cardiovascular diseases (27.3%). Among those not reporting hypertension, 33.4% actually had high blood pressure, which resulted in a final prevalence of hypertension of 78.6% (reported hypertension or measured high blood pressure). Among those not reporting diabetes, only 3.7% had diabetes according the laboratory criteria, with 27.9% as the final prevalence of diabetes (reported diabetes or laboratory criteria). The prevalence of metabolic syndrome was 52.0%. A normal BMI was found in 34.3% of the population, with 57.6% being overweight or obese and 8.1% having a low BMI.

Mean GFR was 75.43±0.75 mL/min/1.73m^2^ (19.6% with GFR ≥ 90, 28.8% with GFR 89–75, 31.8% with GFR 74–60, 14.2% with GFR 59–45, 4.4% with GFR 44–30, 0.8% with GFR 29–15 and only 0.4% with GFR<15 mL/min/1.73m^2^).

GFR < 60 mL/min/1.73m^2^ was present in 19.3% of the individuals, who were older than those with GFR ≥ 60 mL/min/1.73m^2^ (75.3±0.9 versus 69.3±0.7 years, p<0.001). Among those with GFR< 60 mL/min/1.73m^2^ the number of individuals who completed the 8-year basic cycle of schooling was reduced (18.2% versus 30.3%, p = 0.01). Gender, ethnic background, BMI, income, access to private health insurance, perception of health as good or very good and smoking status were similar in both groups. The prevalence of hypertension, diabetes and cardiovascular disease were higher in the decreased GFR group (Table 1).

**Table 1:** Characteristics of the total population and those with GFR < 60 mL/min/1.73 m^2^ and with GFR ≥ 60 mL/min/1.73 m^2^.

	Total	GFR < 60 mL/min/1.73m^2^	GFR ≥ 60 mL/min/1.73m^2^	p-value^a^
		(19.3%)	(80.7%)	
**Age (years)**				<0.001
60–69	54.2%	30.4%	60.1%	
70–79	31.2%	40.0%	29.0%	
80–89	12.5%	23.5%	9.8%	
≥90	2.1%	6.1%	1.2%	
**Females**	60.1%	62.9%	59.4%	0.327
**Caucasians**	58.6%	60.1%	58.2%	0.656
**BMI** ^b^				0.784
low	8.1%	9.4%	7.8%	
normal	34.3%	34.1%	34.3%	
overweight or obese	57.6%	56.5%	57.9%	
**Hypertension** ^c^	78.6%	89.6%	75.9%	<0.001
**Diabetes** ^d^	27.9%	34.4%	26.3%	0.021
**Metabolic syndrome** ^e^	52.0%	56.7%	50.8%	0.095
**Cardiovascular disease** ^f^	27.3%	42.7%	23.5%	<0.001
**No comorbidities** ^g^	11.1%	5.4%	12.4%	0.002

Data were weighted to be representative of the elderly population of São Paulo based on the 2010 Census in Brazil. Data are presented as weighed percentages.

^a^ χ^2^ with Rao-Scott correction.

^b^ BMI: body mass index; low: ≤22 kg/m^2^, normal: 22>BMI>27 kg/m^2^, overweight or obese: ≥27 kg/m^2^.

^c^ Hypertension: self-reported or the mean of three measurements of arterial blood pressure higher than 140/90 mmHg.

^d^ Diabetes mellitus: self-reported or a fasting plasma glucose ≥126 mg/dL or a glycohemoglobin test ≥6.5%.

^e^ Metabolic syndrome: presence of at least three of the following criteria: a waist circumference ≥90 cm for men or ≥80 cm for women, triglycerides ≥150 mg/dL, an HDL cholesterol ≤40 mg/dL for males or ≤50 mg/dL for females, a systolic blood pressure ≥130 mm Hg or a diastolic blood pressure ≥85 mmHg and a fasting plasma glucose ≥100 mg/dL.

^f^ Cardiovascular disease: self-reported.

^g^ No comorbidities: absence of hypertension, diabetes, cardiovascular disease or metabolic syndrome.

**Fig 1:**
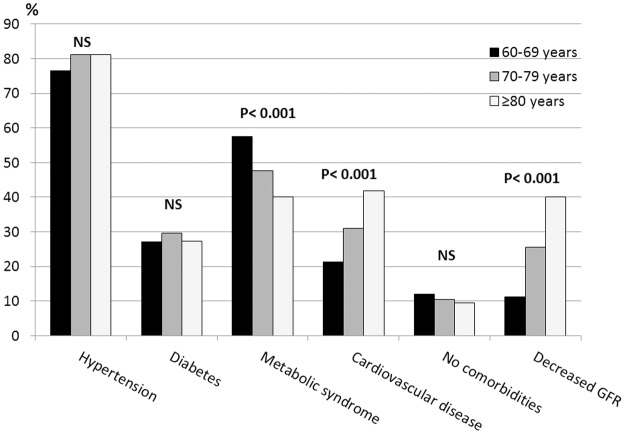
The prevalence of hypertension, diabetes, metabolic syndrome, cardiovascular disease and decreased GFR according to the following age groups: 60–69, 70–79 and ≥80 years. Data were weighted to be representative of the elderly population of São Paulo based on the 2010 Census in Brazil. Data are presented as weighed percentages. Hypertension: self-reported or the mean of three measurements of arterial blood pressure >140/90 mmHg. Diabetes mellitus: self-reported or a fasting plasma glucose ≥126 mg/dL or a glycohemoglobin ≥6.5%. Metabolic syndrome: presence of at least three of the following criteria: a waist circumference ≥90 cm for men or ≥80 cm for women, triglycerides ≥150 mg/dL, an HDL cholesterol ≤40 mg/dL for males or ≤50 mg/dL for females, a systolic blood pressure ≥130 mm Hg or a diastolic blood pressure ≥85 mmHg and a fasting plasma glucose ≥100 mg/dL. Cardiovascular disease: self-reported. None: absence of hypertension, diabetes, metabolic syndrome or cardiovascular disease. Decreased GFR defined as GFR <60 mL/min/1.73 m^2^.

The prevalence of comorbidities and decreased GFR were assessed in the 60–69, 70–79 and ≥ 80 age groups. The prevalence of hypertension (from 76.5 to 81.1%, p = 0.130) and of diabetes (27.1 to 29.7%, p = 0.646) was similar in all groups. The prevalence of metabolic syndrome decreased with age (57.6 to 40.0%, p<0.001), and the prevalence of cardiovascular disease increased with age (21.3 to 41.9%, p<0.001). The prevalence of individuals without any of these diseases was similar in all groups (12.0 to 9.4%, p = 0.777). A sharp increase in the prevalence of decreased GFR was observed with aging: 11.1, 25.5 and 40.1%, respectively (p<0.001) (Fig 1).

The decreased GFR group showed lower levels of hemoglobin (13.9±0.1 vs 14.3±0.1 g/dL, p<0.001) and higher levels of urea (52.6±1.2 vs 35.8±0.4 mg/dL, p<0.001), uric acid (6.1±0.1 vs 5.0±0.1 mg/dL, p<0.001) and calcium (4.54±0.01 versus 4.49±0.01 mEq/L, p = 0.003). Phosphorus, albumin, CRP and ferritin were similar in both groups. Fibrinogen (374±6 versus 341±4 ng/dL, p<0.001), urinary protein:creatinine ratio (0.24±0.05 versus 0.06±0.01 g/g, p<0.001) and presence of renal damage (35.2 versus 14.9%, p<0.001) were higher in the decreased GFR group. The frequency of renal damage increased with the decrease of GFR: 14.0% for GFR ≥ 90 mL/min/1.73m^2^, 15.2% for GFR 89–60 mL/min/1.73m^2^; 23.3% for GFR 59–45 mL/min/1.73m^2^, 24.4% for GFR 44–30 mL/min/1.73m^2^, 44.4% for GFR 29–15 mL/min/1.73m^2^ and 100% for GFR < 15 mL/min/1.73m^2^.

**Fig 2:**
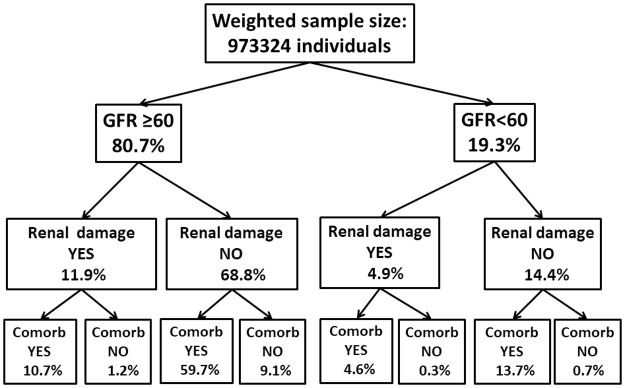
Flow-chart showing the weighted percentage of elderly individuals according to the presence of decreased GFR, renal damage and comorbidities. All the weighted percentages were estimated relative to the entire population, resulting in 100% for each line. Comorb: comorbidities. Decreased GFR defined as GFR <60 mL/min/1.73 m^2^; renal damage defined as presence of proteinuria/hematuria; comorbidities defined as presence of diabetes, hypertension, metabolic syndrome or cardiovascular disease.

Considering the entire population, only 9.5% of the individuals had no concomitant comorbidities or renal damage: 8.8% had GFR ≥ 60 mL/min/1.73m^2^ and 0.7% had GFR < 60 mL/min/1.73m^2^ as shown in Fig 2. In this figure, all weighted percentages were estimated relative to the entire population, resulting in 100% for each line.

However, when only the individuals with GFR <60 mL/min/1.73 m^2^ were analyzed, 3.6% of them had neither renal damage nor associated comorbidities, whereas among those with GFR ≥60 mL/min/1.73 m^2^, 10.9% had none of these conditions.

In the group of individuals with neither renal damage nor associated comorbidities, a comparison between those with GFR <60 mL/min/1.73 m^2^ (weighted sample size: 6848) and those with GFR ≥60 mL/min/1.73 m^2^ (weighted sample size: 88,662) showed that the former individuals were older (58.0% versus 3.9% aged ≥ 80 years, p<0.001), were mainly female (87.5% versus 42.3%, p = 0.004) and Caucasian (95.1% versus 69.7%, p = 0.023). Body mass index and laboratory test results were similar in both groups, except for lower hemoglobin levels in those with GFR <60 mL/min/1.73 m^2^ (12.8±0.2 versus 14.6±0.2 g/dL, p<0.001).

The logistic regression showed that increasing age and presence of hypertension or cardiovascular disease were associated with decreased GFR. (Table 2)

**Table 2:** Association of age and presence of comorbidities with the finding of a decreased GFR.

	Estimate^a^	aOR^b^	95% Confidence Interval	p-value
**Age (years)**				
60–69		1	**--**	
70–79	0.86	2.36	1.44–3.89	0.001
≥80	1.51	4.52	2.68–7.61	<0.001
**Cardiovascular disease**^c^	0.62	1.86	1.31–2.64	<0.001
**Hypertension**^d^	0.83	2.29	1.32–3.96	0.004
**Diabetes**^e^	0.36	1.43	0.95–2.15	0.090

^a^ Logistic regression model, adjusted for gender, schooling and minimum wage by month. Decreased GFR defined as GFR <60 mL/min/1.73 m^2^

^b^ adjusted odds ratio.

^c^ Cardiovascular disease: self-reported.

^d^ Hypertension: self-reported or the mean of three measurements of arterial blood pressure >140/90 mmHg.

^e^ Diabetes mellitus: self-reported or a fasting plasma glucose ≥126 mg/dL or a glycohemoglobin ≥6.5%.

## Discussion

Glomerular filtration rate decreases with aging, but studies examining whether this process is physiological or pathological have yielded conflicting results. [23–25] To the best of our knowledge, our study was the first that was designed to address the following question: how many elderly individuals have a decreased GFR without renal damage or concomitant comorbidities? We showed that only 0.7% of the population aged ≥ 60 years fit this clinical condition. Among those with GFR<60 mL/min/1.73 m^2^, the proportion of individuals without renal damage or associated comorbidities was more than three-fold less than among those with GFR ≥ 60 mL/min/1.73 m^2^ without these associated conditions. The presence of a GFR<60 mL/min/1.73 m^2^ was likely not associated with undernutrition or muscle wasting because BMI values were similar to those among individuals with GFR ≥60 mL/min/1.73 m^2^. However, both increased age and presence of comorbidities were independent factors associated with decreased GFR. These data suggest that a finding of a decreased GFR in elderlies is frequently associated to a pathological condition instead of only a normal aging process.

Consistent with our findings, recent data from the “Medicare 5 percent sample survey” showed a 10.6% CKD prevalence among individuals aged ≥ 65 years, but only 1.8% of these individuals had no concomitant diabetes or cardiovascular disease. [2] This survey assessed a large databank but only analyzed diabetes and cardiovascular diseases as comorbidities and used a code for CKD diagnosis, which has lower sensitivity than the assessment of GFR. Even the Berlin Initiative Study (BIS) which analyzed the risk factors for decreased GFR and albuminuria did not reported the prevalence of individuals with GFR<60 mL/min/1.73 m^2^ without comorbidities. [26]

The Kidney Disease: Improving Global Outcomes (KDIGO) practice guidelines define CKD as a GFR < 60 ml/min/1.73m^2^ or the presence of kidney damage (abnormal urinalysis, kidney imaging, or renal biopsy) that persists for ≥ 3 months.[27] However, most of the population-based studies that assessed CKD prevalence are based on only one GFR measurement.[28–31] In this study, we considered a GFR < 60 ml/min/1.73m2 as decreased GFR (not CKD) and an abnormal urinalysis or proteinuria as renal damage.[8]

Epidemiological population-based data on CKD prevalence are scarce, particularly in low and middle-lower income countries where the elderly population is increasing, as is the prevalence of diabetes, hypertension and obesity.[32] Most of the population-based studies on CKD prevalence have evaluated adults in general and have shown a strong positive influence of age on CKD prevalence in several countries, such as China, the UK, Ireland, the Netherlands and the USA.[28–30,33] In a recent adult population-based study in Iran the prevalence of CKD was 23.7%. The major risk factors for CKD were obesity or overweight, diabetes, hypertension and dyslipidemia. [34]

The few population–based studies designed specifically to assess the elderly population have shown great diversity with respect to CKD prevalence, ranging from 23.4 to 58.5%.[1] This dissimilarity may be partially explained by the use of different equations to estimate GFR (there is no consensus as to which one is the best equation for the elderly). In the Berlin Initiative Study (BIS) which included 2069 individuals aged ≥ 70 years the prevalence of GFR <60 mL/min/1.73m^2^ varied from 37.1% to 61.7% according to the equation used to estimate GFR: the lowest value was obtained with MDRD equation and the highest with BIS1. [26] Also an Italian study of 800 individuals aged 85 or older designed to test several equations to estimate GFR showed wide variation in the prevalence of decreased GFR: 90.7% with the Cockcroft-Gault equation, 84.4% with the BIS, 53.6% with CKD-EPI (Chronic Kidney Disease Epidemiology Collaboration), 48.1% with MDRD and 23.3% with the Mayo Clinic equation. The authors suggested that the MDRD equation, which we used, was the most consistent predictor of 5-year mortality. [35] The prevalence of decreased GFR that we observed in our study (19.6%) is similar to that reported in a Canadian study (18.6%), which included individuals aged ≥ 65 years and that used the MDRD equation and a single GFR estimation. [31] The GFR estimated by CKD-EPI, using our non-calibrated SCr values, gave results almost identical to those we found using the simplified MDRD equation: 19.7% with decreased GFR by MDRD equation and 21.9% by CKD-EPI equation (data not shown).

We observed a higher prevalence of decreased GFR than two other population-based Brazilian studies. [10–11] The Bambui study observed a prevalence of increased SCr (single measurement) of 5.1%, whereas our study found a prevalence of 16.2% (data not shown). This discrepancy may be partially explained by the characteristics of the two populations: age (69.3±7.4 in the Bambui study versus 73.1±9.5 years in our study), differences in laboratory methods (the normal upper limit for SCr was higher for females in the Bambui study) and striking differences in the characteristics of the two cities (Bambui is a small rural city, and São Paulo is the biggest megalopolis in South America).[10,36] A positive association between a decreased GFR and an urban dwelling has been reported in adult population.[27, 34] The Tubarão study found a prevalence of decreased GFR of 13.6% (single GFR, CKD-EPI equation). As with the Bambui study, in the Tubarão study the mean age was smaller (68.6±7 years), and Tubarão is also a smaller city (97,235 inhabitants) than São Paulo (11,253,000 inhabitants in 2010).[11]

Our study has some limitations. Since only once the renal function was evaluated, we couldn´t classify, according to KDIGO guidelines, the individuals who had GFR <60 mL/min/1.73m^2^ as having CKD. [6] We used the simplified MDRD equation to estimate the GFR because our SCr measurement was not calibrated (in this case the equation has an accuracy of 74%). [37–38] We didn’t have access, for economic reasons, to the calibrated SCr used in the newer equations or to albuminuria to classify renal damage. However, the laboratory methods used in the present study are the most used methods for SCr and proteinuria dosage in our country and thus our data represent what the doctors in Brazil have to deal with. The cutoff of GFR <60 mL/min/1.73m2 to diagnose decreased GFR may result in an error of approximately 9%. [39] We did not assess other possibilities that may be related to decreased GFR (*e*.*g*., use of nephrotoxic drugs), although we assessed a larger number of comorbidities than previous studies.

Our study has important strengths. It was specifically designed to address the following question: “is the frequency of decreased GFR in the elderly attributable to only the physiological aging process?” Our study was also designed to obtain a representative sample of the elderly population of São Paulo at the time the study was conducted. However, the individuals that were not included in the renal damage evaluation due to difficulty collecting urine in this setting were slightly older and had fewer years of schooling. The diagnoses of decreased GFR and renal damage were based on actual serum and urine measurements instead of codes or retrospective data. We used anthropometric and laboratory measurements in addition to questionnaires when analyzing comorbidities.

In conclusion, in the São Paulo megalopolis, the elderly have a high prevalence of decreased GFR, which is rarely present without concomitant kidney damage and/or chronic diseases that may affect the kidneys. These data suggest that a decreased GFR in the elderly is strongly indicative of the presence of concomitant renal disease, which should be investigated.

## Supporting information

S1 File Set of data analysed for each individual included in this study. (XLSX)
